# Cohort Profile: The Children’s Health in London and Luton (CHILL) cohort

**DOI:** 10.1093/ije/dyaf055

**Published:** 2025-05-29

**Authors:** Helen E Wood, Rosamund E Dove, Jasmine Chavda, Grainne Colligan, Louise Cross, Harpal Kalsi, James Scales, Ivelina Tsocheva, Sean Beevers, Bill Day, Monica Fletcher, W James Gauderman, Jonathan Grigg, Hajar Hajmohammadi, Frank J Kelly, Borislava Mihaylova, Chris Newby, Gurch Randhawa, Aziz Sheikh, Veronica Toffolutti, Florian Tomini, Esther van Sluijs, Ian S Mudway, Chris J Griffiths

**Affiliations:** Wolfson Institute of Population Health, Faculty of Medicine and Dentistry, Queen Mary University of London, London, UK; Centre for Applied Respiratory Research, Innovation, and Impact, Nuffield Department of Primary Care Health Sciences, University of Oxford, Oxford, UK; Wolfson Institute of Population Health, Faculty of Medicine and Dentistry, Queen Mary University of London, London, UK; Centre for Applied Respiratory Research, Innovation, and Impact, Nuffield Department of Primary Care Health Sciences, University of Oxford, Oxford, UK; Institute for Health Research, University of Bedfordshire, Luton, UK; Wolfson Institute of Population Health, Faculty of Medicine and Dentistry, Queen Mary University of London, London, UK; Wolfson Institute of Population Health, Faculty of Medicine and Dentistry, Queen Mary University of London, London, UK; Wolfson Institute of Population Health, Faculty of Medicine and Dentistry, Queen Mary University of London, London, UK; Wolfson Institute of Population Health, Faculty of Medicine and Dentistry, Queen Mary University of London, London, UK; Centre for Applied Respiratory Research, Innovation, and Impact, Nuffield Department of Primary Care Health Sciences, University of Oxford, Oxford, UK; Institute for Health Research, University of Bedfordshire, Luton, UK; MRC Centre for Environment and Health, Imperial College London, London, UK; Centre for Applied Respiratory Research, Innovation, and Impact, Nuffield Department of Primary Care Health Sciences, University of Oxford, Oxford, UK; Centre for Applied Respiratory Research, Innovation, and Impact, Nuffield Department of Primary Care Health Sciences, University of Oxford, Oxford, UK; Nuffield Department of Primary Care Health Sciences, University of Oxford, UK; Division of Biostatistics, Department of Population and Public Health Sciences, University of Southern California, Los Angeles, USA; Centre for Applied Respiratory Research, Innovation, and Impact, Nuffield Department of Primary Care Health Sciences, University of Oxford, Oxford, UK; Blizard Institute, Faculty of Medicine and Dentistry, Queen Mary University of London, London, UK; Wolfson Institute of Population Health, Faculty of Medicine and Dentistry, Queen Mary University of London, London, UK; Centre for Applied Respiratory Research, Innovation, and Impact, Nuffield Department of Primary Care Health Sciences, University of Oxford, Oxford, UK; MRC Centre for Environment and Health, Imperial College London, London, UK; Wolfson Institute of Population Health, Faculty of Medicine and Dentistry, Queen Mary University of London, London, UK; Centre for Applied Respiratory Research, Innovation, and Impact, Nuffield Department of Primary Care Health Sciences, University of Oxford, Oxford, UK; Nuffield Department of Population Health, University of Oxford, Oxford, UK; School of Medicine, University of Nottingham, Nottingham, UK; Institute for Health Research, University of Bedfordshire, Luton, UK; Centre for Applied Respiratory Research, Innovation, and Impact, Nuffield Department of Primary Care Health Sciences, University of Oxford, Oxford, UK; Nuffield Department of Primary Care Health Sciences, University of Oxford, UK; Wolfson Institute of Population Health, Faculty of Medicine and Dentistry, Queen Mary University of London, London, UK; Wolfson Institute of Population Health, Faculty of Medicine and Dentistry, Queen Mary University of London, London, UK; MRC Epidemiology Unit, University of Cambridge, Cambridge, UK; MRC Centre for Environment and Health, Imperial College London, London, UK; Wolfson Institute of Population Health, Faculty of Medicine and Dentistry, Queen Mary University of London, London, UK; Centre for Applied Respiratory Research, Innovation, and Impact, Nuffield Department of Primary Care Health Sciences, University of Oxford, Oxford, UK; Nuffield Department of Primary Care Health Sciences, University of Oxford, UK

**Keywords:** cohort, air pollution, children, lung function, natural experiment, health impacts

Key Features

- The Children’s Health in London and Luton (CHILL) cohort was established to investigate the impact of London’s Ultra Low Emission Zone (ULEZ) on children’s health. Key strengths of CHILL include: the parallel prospective cohort, natural experimental design in which children living in London (exposed to the ULEZ) and children living in Luton (not exposed to a ULEZ or other major air-pollution control measure) are followed over time and compared; its large size (compared with similar studies) and ethnic diversity; high-resolution air pollution exposure data; and objective physiological measurements of lung function.
- In total, 3414 participants (aged 6–9 years; 52% female) were recruited in central London (intervention site, *n* = 1664) and Luton (comparator site, *n* = 1750). Baseline health assessments took place in 2018–19, prior to implementation of the ULEZ, with four annual follow-up assessments.
- Attrition ranged from 6.9% in the first follow-up year to 33.8% in the final year, with 1590 participants remaining after 5 years (*n* = 754 London, *n* = 836 Luton).
- Data were collected by using questionnaires, health assessments, and physical-activity monitoring. Study outcomes assessed include lung-function growth, physical activity, transport use, cognitive function and mental health indicators, healthcare utilization, personal and socioeconomic health determinants, and air-pollution exposures.
- The CHILL study welcomes collaborations and will consider all reasonable requests for data access. Requests, including a description of planned projects, should be sent to the corresponding author CHILL@qmul.ac.uk at Queen Mary University of London, London, UK.

## Why was the cohort set up?

Air pollution contributes to >8.1 million annual deaths worldwide and poses the largest environmental risk to health [1, 2]. This burden disproportionately affects the most disadvantaged and vulnerable members of society [3]. In Europe, nearly 800 000 excess deaths per year are attributed to poor air quality in densely populated urban areas [4] while, in the UK, most under-5s, young adults, and poorer households live in areas with the highest concentrations of traffic-related air pollution (TRAP) [5]. As their lungs are still developing, children are particularly sensitive to the harmful effects of air pollution [6] and concern has increased over recent years that long-term exposures can cause developmental delays with consequences to children’s health across the life course [7]. Exposure to TRAP has been associated with reduced lung function in primary-school children in London [8], Europe [9], and the USA [10]. Failure to achieve maximal lung-function growth by early adulthood is associated with increased risk of chronic respiratory disease and early mortality [11].

The imperative to reduce children’s exposure to air pollution [12], particularly in disadvantaged communities, is reflected in stringent clean air targets that were recently updated by the World Health Organization [13]. Clean air zones (CAZs), in which traffic control measures are employed to improve air quality, are an important tool in the effort to meet these targets and are being implemented in cities around the world [14]. The ultra low emission zone (ULEZ) in London is a CAZ that penalizes the entry of high-polluting vehicles (based on European class emission standards—see Supplementary Figure S1) and was introduced in 2019 with the aim of improving air quality and public health [15].

The Children’s Health in London and Luton (CHILL) cohort was established in 2018–19 to assess the impact of the ULEZ on children’s health and development by using a prospective parallel cohort design. The primary objective of the study is to investigate the impact of the ULEZ on lung-function growth. Secondary objectives will consider the impact of the ULEZ on air quality, respiratory health, physical activity, travel habits, health-related quality of life, and health costs not related to the National Health Service (NHS). Sub-studies undertaken alongside the CHILL study will investigate:

- the impact of air quality on cognitive development and mental health;
- the impact of air quality on epigenetics;
- exposure to heavy metals from TRAP;
- value for money of the ULEZ by undertaking a cost–consequence analysis.

**Figure 1.**
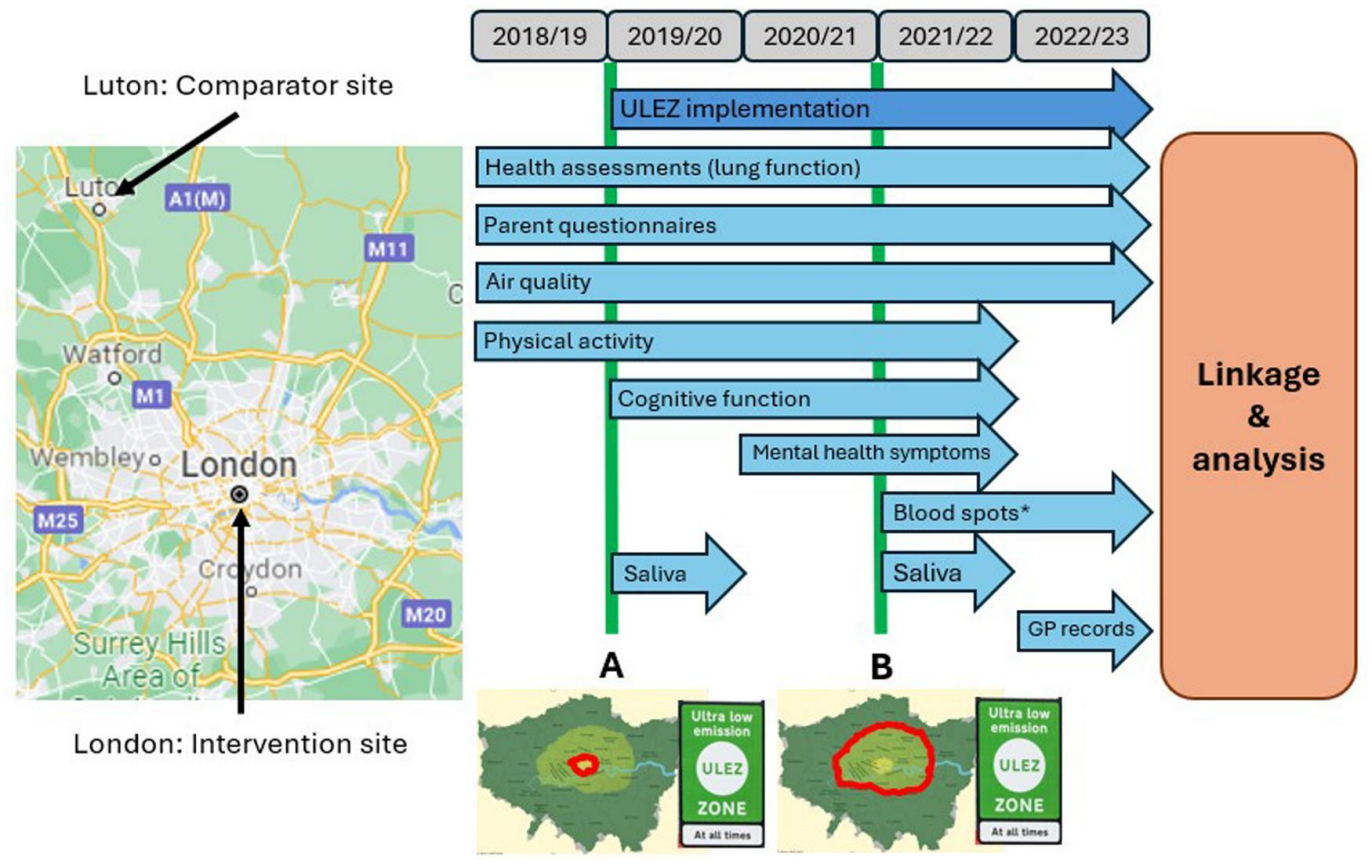
Study overview. Health outcome data were collected annually, beginning in the 2018/19 academic year (prior to implementation of the ULEZ) and continuing for 4 follow-up years until 2022/3. The thick vertical lines indicate the implementation of the initial ULEZ area in central London (A) and the first expansion of the scheme to cover 380 km^2^ of inner London (B) (outlined on corresponding maps). GP, general practice (primary care). *Participants provided blood-spot samples in either the third or fourth follow-up health assessment.

The CHILL study protocol has been published elsewhere [16]. An overview of the timeline and data collection is provided in Figure 1. The CHILL study was supported by a National Institute for Health Research (NIHR) Public Health Research grant (number 16/139/09) with additional funding for sub-studies by the NIHR Collaboration for Leadership in Applied Health Research (CLAHRC) North Thames, Barts Charity, The Mayor of London, and the NIHR Applied Research Collaboration (ARC) North Thames.

## Who is in the cohort?

The cohort comprises children who are attending primary schools within or on the boundary of the initial ULEZ area in central London (see Figure 1) or in the Luton/Dunstable area (referred to as “Luton” for simplicity). Luton was selected as the comparator site due to its similarity to central London in terms of population demographics and mixture of local air-pollutant sources, while having no plans to introduce any form of CAZ and being sufficiently distant from London to minimize contamination from the ULEZ [17]. Ethical approval for the study was given by Queen Mary University of London Ethics of Research Committee (2018/08) and NHS West of Scotland Research Ethics Committee 4 (22/WS/0065). Written informed consent was obtained from the participants’ parent/carer. Participants were also asked for their verbal assent.

All state primary schools in the study areas were approached, with 44 schools in central London and 40 in Luton (67% and 71% of those invited, respectively) agreeing to participate. We invited children in school Years 2, 3, and 4 (aged 6–9 years) to take part, except in large schools (more than three classes per year group), in which only children in Year 3 were invited. Study packs were delivered to parents in children’s school bags (including information sheets, a consent form, and a parental questionnaire). Sample-size requirements [16] necessitated a target of 3200 participants (1600 each in London and Luton) and 40 schools from each site. Of the children invited, signed consent forms were returned by 1664 in London (37%) and 1750 in Luton (36%). Children participating in the main study were also invited to take part in the CHILL sub-studies. Recruitment is summarized in Supplementary Figures S2 and S3.

Children can perform spirometry from age 6 years [18], hence we recruited those aged 6–9 years at baseline to allow 2–3 years of follow-up within primary schools (to age 11 years). Initially, we did not intend to follow up participants after they had moved to secondary school; however, due to the disruption caused by the COVID-19 pandemic, we added a fourth year of follow-up for all participants, including those attending secondary schools.

## How often have they been followed up?

Four annual follow-up measurements have been taken since the baseline in 2018/19 (see Figure 1). Participation and attrition rates are provided in Table 1. Attrition was low in the first 2 years of follow-up (6.9% and 3.4%), but increased thereafter (21.8% and 33.8%), driven mainly by the movement of children from primary to secondary school. We were not able to trace some children once they had left primary school or visit all secondary schools to which participants had moved. Children who were lost to follow-up were on average slightly older than those who were retained throughout the study, reflecting the increased attrition of those moving to secondary school. Otherwise, their characteristics were similar (see Supplementary Table S1).

**Table 1.** Participation in the main CHILL study and sub-studies by year of data collection. Values are *n* (% of total recruited/retained), unless otherwise stated.

Variable	Baseline	FY1	FY2	FY3	FY4
Recruited/retained (*n*)	3414	3177	3070	2401	1590
Cohort attrition (by year) (%)	–	6.9	3.4	21.8	33.8
Cohort attrition (cumulative) (%)	–	6.9	10.1	29.7	53.4
Present at school for health assessment	3317 (97.2)	2561 (80.6)	527 (17.2)	2161 (90.0)	1466 (92.2)
Declined to participate (*n*)	6	1	1	13	13
Height and weight measured^a^	3311 (99.8)	2559 (99.9)	525 (99.6)	2141 (99.1)	1453 (99.1)
Attempted spirometry^a^	3243 (97.8)	2535 (99.0)	526 (99.8)	2133 (98.7)	1441 (98.3)
Post-bronchodilator FEV_1_ assessed^b^	2619 (80.8)	2096 (82.7)	399 (75.9)	1690 (79.2)	1192 (82.7)
Post-bronchodilator FVC assessed^b^	2577 (79.5)	2051 (80.9)	393 (74.7)	1603 (75.2)	1086 (75.4)
Parent questionnaire completed	3143 (92.1)	2223 (70.0)	1187 (38.7)	1280 (53.3)	772 (48.6)
Physical activity (*n*)	2727	2329	409	1212	–
GPS sub-study (*n*)	710	696	76	509	–
Cognitive function sub-study (*n*)	–	818	1324	1002	–
Mental health sub-study (*n*)	–	–	929	745	–
Epigenetics sub-study (saliva sample) (*n*)	–	1251	–	323	–
Heavy-metals sub-study (*n*)	–	–	–	432	127

FY, follow-up year; FEV_1_, forced expiratory volume in 1 second; FVC, forced vital capacity; GPS, global positioning system; cohort attrition is proportion of previous year’s participants who were lost to follow-up.

a Percentage of participants present at school for health assessment.

b Percentage of participants who attempted spirometry.

During the second follow-up year (2020–1), our data collection was significantly shortened (to 4 months, April–July 2021) due to the COVID-19 pandemic.

Annual parental questionnaire returns decreased over time, from 92.1% at baseline to 48.6% in the final follow-up year (Table 1). Returns were particularly low (38.7%) for the second follow-up year, in which questionnaires were mailed to participants due to the COVID-19 pandemic.

## What has been measured?

### Main study

Data were collected directly from children via health assessments, bio-samples, accelerometry, and questionnaires, and indirectly via questionnaires completed by their parents and teachers.

Annual health assessments took place at the children’s schools and were performed by trained CHILL team members. Height, seated height, and weight were measured, and children completed a verbal questionnaire regarding asthma, use of inhalers, and their mode of transport to school. Lung function was assessed via pre- and post-bronchodilator spirometry, using Vitalograph 6000 Alpha Touch Spirometers (Vitalograph, Buckingham, UK) and following American Thoracic Society and European Respiratory Society guidelines [19]. Recorded measurements included forced expiratory volume in one second (FEV_1_), forced vital capacity (FVC), FEV_1_/FVC ratio, peak expiratory flow, and maximal mid-expiratory flow between 75% and 25% of the FVC. Parents were asked to complete an annual questionnaire coincidently with the health assessment (see Supplementary material).

### Sub-studies

To assess physical activity, participants were given Actigraph triaxial accelerometers (Actigraph wGT3X-BT, Pensacola, Florida, USA) to wear for the week following the health assessment. A subset of participants were also given GPS monitors (QStarz BT-Q1000XT, Taipei, Taiwan). Physical-activity data were collected at baseline and during the first three annual follow-up health assessments (see Figure 1).

Saliva samples were collected at the first and third follow-up assessments, using Oragene-DNA collection kits (OG-DNA). DNA methylation analysis will be performed by using the Infinium MethylationEPIC array to identify epigenetic signatures associated with short- and long-term air-pollution exposures.

Finger-prick blood samples were collected at the third or fourth follow-up assessments and will be used to analyse heavy-metal exposures (lead, arsenic, and cadmium) via inductively coupled plasma mass spectrometry.

Cognitive function and mental health symptoms were assessed at additional school visits between November 2019 and July 2022 (see Figure 1 and Supplementary material). Cognitive function measurements were obtained via interactive tasks performed on tablet computers (Lenovo ThinkPad Helix type 20CG or 20CH) by using a digital stylus or finger touch for input [20]. For the assessment of mental health symptoms, children completed the Revised Children’s Anxiety and Depression questionnaire (validated for self-completion for 8- to 18-year-olds) [21] and their class teachers completed the Strengths and Difficulties questionnaire.

To assess the cost-effectiveness of the ULEZ in terms of healthcare utilization, primary-care records and hospital-episode statistics will be collected for participants with specific consent (requested at recruitment and confirmed in the final year of the study; see Supplementary material).

Outcomes from the sub-studies will be published separately.

### Air quality

To assess the impact of air quality on all study metrics, monthly and annual modeled air-pollutant exposures at the level of individual participant home addresses will be estimated for the criterion pollutants [i.e. those with legal standards—nitrogen dioxide (NO_2_), ozone (O_3_), particulate matter with an aerodynamic diameter of <10 microns (PM_10_) and <2.5 microns (PM_2.5_); see Supplementary material].

## What has it found?

### Demographic characteristics

The characteristics of the London and Luton cohorts are shown in Table 2. Overall, slightly more girls were recruited than boys (52.1% vs. 47.9%), with the difference in proportions being more marked for London than for Luton. Differences were observed in the ethnicity of the two cohorts, with higher proportions of White and Asian/Asian British children recruited in Luton and higher proportions of Black/Black British and Mixed children recruited in London, reflecting the underlying populations (see Supplementary Tables S2A and S2B) [22, 23]. Height, weight, and body mass index (BMI) were similar between the two cohorts at baseline. The Index of Multiple Deprivation (IMD) was lower in London than in Luton (3.59 ± 1.70 vs. 4.03 ± 2.06), indicating higher deprivation in London (see Supplementary Figure S4). Age, sex, ethnicity, BMI, and IMD will be included as covariates in the final analysis.

**Table 2.** Characteristics of the cohort at baseline for all participants and by site. Data are presented as mean and standard deviation for continuous variables and as frequency and percentage for categorical variables.

Variable	All	London	Luton
Total participants (*n*)	3414	1664	1750
Mean (SD) age (years)	7.87 (0.85)	7.95 (0.89)	7.80 (0.80)
Female [*n* (%)]	1779 (52.1)	919 (55.2)	860 (49.1)
School year [*n* (%)]			
Year 2 (age 6–7 years)	961 (28.1)	484 (29.1)	477 (27.3)
Year 3 (age 7–8 years)	1480 (43.4)	604 (36.3)	876 (50.1)
Year 4 (age 8–9 years)	973 (28.5)	576 (34.6)	397 (22.7)
Mean (SD) height (cm)	128.4 (7.7)	129.3 (8.1)	127.6 (7.4)
Mean (SD) weight (kg)	28.5 (7.0)	29.0 (7.3)	28.0 (6.8)
Mean (SD) BMI (kg/m^2^)	17.1 (2.8)	17.2 (2.9)	17.0 (2.8)
Mean (SD) deprivation index	3.82 (1.91)	3.59 (1.70)	4.03 (2.06)
Ethnicity [*n* (%)]			
White	1159 (33.9)	496 (29.8)	663 (37.9)
Asian/Asian British	1048 (30.7)	369 (22.2)	679 (38.8)
Black/Black British	449 (13.2)	317 (19.1)	132 (7.5)
Mixed	366 (10.7)	224 (13.5)	142 (8.1)
Other	186 (5.4)	140 (8.4)	46 (2.6)
Missing	206 (6.0)	118 (7.1)	88 (5.0)
Participated in health assessment [*n* (%)]	3311 (97.0)	1606 (96.5)	1705 (97.4)
Self-reported at health assessment [*n* (%)]^a^			
Asthma	361 (10.9)	146 (9.1)	215 (12.6)
Inhaler use	444 (13.4)	197 (12.3)	247 (14.5)
Blue inhaler use^b^,^e^	414 (93.2)	183 (92.9)	231 (93.5)
Brown inhaler use^b^,^f^	128 (28.8)	61 (31.0)	67 (27.1)
Other inhaler use^b^	39 (8.8)	22 (11.2)	17 (6.9)
Travelled to school that day by walking^c^	2012 (60.8)	1069 (66.6)	943 (55.3)
Travelled to school that day by scooting^c^	144 (4.3)	114 (7.1)	30 (1.8)
Travelled to school that day by bike^c^	62 (1.9)	43 (2.7)	19 (1.1)
Travelled to school that day by private car^c^	976 (29.5)	200 (12.5)	776 (45.5)
Travelled to school that day by taxi^c^	22 (0.7)	7 (0.4)	15 (0.9)
Travelled to school that day by bus^c^	231 (7.0)	207 (12.9)	24 (1.4)
Travelled to school that day by train/underground^c^	53 (1.6)	53 (3.3)	0 (0.0)
Returned completed parent questionnaire [*n* (%)]	3143 (92.1)	1486 (89.3)	1657 (94.7)
Reported from parent questionnaire [*n* (%)]^d^			
Cigarette smoker in the household	700 (22.3)	264 (17.8)	436 (26.3)
Child had wheezing in last 12 months	357 (11.4)	162 (10.9)	195 (11.8)
Child had severe wheeze	145 (4.6)	65 (4.4)	80 (4.8)
Child ever had asthma	377 (12.0)	150 (10.1)	227 (13.7)
Child experienced exercise-induced wheeze in last 12 months	239 (7.6)	107 (7.2)	132 (8.0)
Child had dry cough at night in last 12 months, not associated with a cold	626 (19.9)	303 (20.4)	323 (19.5)
Child had problem with sneezing or runny/blocked nose in last 12 months, not associated with a cold	680 (21.6)	368 (24.8)	312 (18.8)
Child also had itchy-watery eyes in the last 12 months	359 (11.4)	196 (13.2)	163 (9.8)
Child ever had hay fever	888 (28.3)	435 (29.3)	453 (27.3)
Child had eczema in last 12 months	423 (13.5)	215 (14.5)	208 (12.6)
Child ever had eczema	868 (27.6)	426 (28.7)	442 (26.7)
Child had current wheeze, rhinitis, and eczema	69 (2.2)	40 (2.7)	29 (1.8)

BMI, body mass index.

a Percentage of those who participated in the health assessment.

b Percentage of those who reported inhaler use.

c Percentage totals add to >100 because more than one answer could be given.

d Percentage of those who returned a completed parent questionnaire.

e Blue inhaler is a rescue inhaler (taken in response to symptoms), usually salbutamol or Ventolin.

f Brown inhaler is an inhaled corticosteroid (preventative), usually beclomethasone.

### Air quality

The mean annual air-pollution concentrations at the roadside and urban background sites within the ULEZ and in Luton for 2018 and 2019 are given in Table 3 (Supplementary Table S3 provides details of specific monitoring data used) [24–28]. Roadside NO_2_ concentrations (within 1–5 m of a busy road) were markedly higher within the ULEZ than in Luton, but this difference was strongly influenced by the kerbside sites (within 1 m of the kerb of a busy road) included in the London measurements, particularly Marylebone Road. Kerbside data were not available for Luton. Background NO_2_ data from an automated network were not available for Luton, but data from diffusion-tube measurements [28] indicated that background NO_2_ concentrations were also higher within the ULEZ than in Luton, albeit by a smaller margin than at the roadside.

**Table 3.** Measured annual mean pollutant concentrations from selected automatic air-quality-monitoring sites.^a^ Data are presented as mean and SD. The *N* value is the number of monitoring sites included in mean.

Pollutant	Measurement	2018		2019	
	location	London—ULEZ	Luton	London—ULEZ	Luton
NO_2_ (µg/m^3^)	Roadside	60.6 ± 18.2 (*n* = 5)	40.0 ± 4.2 (*n* = 2)	46.6 ± 10.4 (*n* = 6)	39.5 ± 0.7 (*n* = 2)
	Urban background	34.5 ± 3.7 (*n* = 4)	25.7 ± 5.9	33.8 ± 3.9 (*n* = 4)	26.2 ± 5.9
PM_2.5_ (µg/m^3^)	Roadside	13.1 ± 4.1 (*n* = 2)	10.0 (*n* = 1)	11.6 ± 3.5 (*n* = 2)	10.0 (*n* = 1)
	Urban background	10.5 ± 0.7 (*n* = 2)	NA	11.5 ± 0.7 (*n* = 2)	NA
PM_10_ (µg/m^3^)	Roadside	25.2 ± 2.7 (*n* = 5)	16.0 (*n* = 1)	24.0 ± 2.1 (*n* = 5)	16.0 (*n* = 1)
	Urban background	18.0 ± 1.7 (*n* = 3)	17.0 (*n* = 1)	17.3 ± 0.6 (*n* = 3)	15.0 (*n* = 1)

NO_2_, nitrogen dioxide; PM_10_, particulate matter with an aerodynamic diameter of <10 microns; PM_2.5_, particulate matter with an aerodynamic diameter of <10 microns.

a Urban background NO_2_ data for Luton are from diffusion-tube measurements; NA, data not available. Data sources: [26–30].

Higher roadside particulate concentrations were also observed within the ULEZ compared with Luton, although these differences were less pronounced. However, background PM_10_ concentrations were similar at the two locations. Background PM_2.5_ measurements were not available for Luton. Modeled data for NO_2_, PM_2.5_, and PM_10_, for both sites are provided in the Supplementary materials (Supplementary Figure S5) [29].

### Health outcomes

Children’s self-reported asthma and inhaler use were similar between London and Luton at baseline, while exposure to second-hand cigarette smoke was higher in Luton than in London (17.8% vs. 26.3%) (Table 2). Parent-reported respiratory symptoms (Table 2), health-related quality of life (Supplementary Table S4), and non-NHS health-related costs (Supplementary Tables S5 and S6) were similar between London and Luton.

Children’s self-reported mode of travel to school differed markedly between the two sites, with far more children travelling to school via active modes (walking, scooting, or cycling) or public transport in London compared with in Luton (active: 76.4% vs. 58.2%, public: 16.2% vs. 1.4%). Conversely, far more children travelled by private car or taxi in Luton (12.9% vs. 46.4%).

At baseline, 96.2% of participants (*n* = 3283) attempted spirometry, with most providing successful measurement of the primary outcome variable (post-bronchodilator FEV_1_; 76.7%, *n* = 2619). Those who did not attempt it were either absent, unable, or unwilling to undertake spirometry. Successful pre- and post-bronchodilator FEV_1_ was achieved for 71.8% of participants (*n* = 2452), permitting the calculation of reversibility.

Spirometry variables are given as absolute values and as percentages of the predicted value (see Table 4) based on age, height, weight, and assigned ethnic population, as categorized by using the Global Lung Initiative [30]. Mean absolute lung function values were similar between London and Luton (FEV_1_: 1.57 ± 0.30 vs. 1.57 ± 0.29 L; FVC: 1.79 ± 0.35 vs. 1.81 ± 0.34 L).

**Table 4.** Post-bronchodilator spirometry measurements at baseline for the whole cohort and by site. Data are presented as mean and standard deviation.

Variable	All	London	Luton
FEV_1_ (L)	1.57 (0.30)	1.57 (0.30)	1.57 (0.29)
FEV_1_ (% predicted)^a^	102.0 (13.4)	101.8 (13.2)	102.2 (13.5)
FVC (L)	1.79 (0.35)	1.79 (0.35)	1.81 (0.34)
FVC (% predicted)^a^	107.8 (13.5)	106.2 (13.5)	109.2 (13.5)
FEV_1_/FVC (%)	87.6 (6.2)	87.9 (6.2)	87.2 (6.3)
PEF (L)	219.2 (50.1)	220.8 (51.2)	217.8 (49.0)
PEF (% predicted)^a^	115.2 (24.0)	114.2 (23.0)	116.1 (24.9)
MMEF (L)	2.09 (0.54)	2.10 (0.55)	2.08 (0.53)
MMEF (% predicted)^a^	108.7 (25.6)	108.7 (25.2)	108.8 (25.9)
Change^b^ in FEV_1_ (mL)	74.5 (123.7)	79.0 (126.3)	70.3 (121.1)
Change^b^ in FEV_1_ (% change)^c^	5.35 (9.05)	5.70 (9.37)	5.03 (8.72)
Change^b^ in FVC (mL)	31.0 (93.2)	32.0 (96.8)	30.0 (89.7)
Change^b^ in FVC (% change)^c^	1.94 (5.57)	2.01 (5.71)	1.87 (5.43)

FEV_1_, forced expiratory volume in 1 second; FVC, forced vital capacity; PEF, peak expiratory flow; MMEF, maximal mid-expiratory flow; change in FEV_1_.

a Percentage of predicted value based on age, height, weight, and assigned ethnic population, as categorized by using the Global Lung Initiative [31].

b From pre- to post-bronchodilator.

c Percentage change in absolute value (mL).

In summary, the CHILL cohort comprises a large sample of ethnically diverse children living in moderately disadvantaged areas of London and Luton with high levels of TRAP. The cohort is well matched for high-quality pre-intervention lung-function data and demographic variables, with parallel high-quality measurement/modeling of air quality.

## What are the main strengths and weaknesses?

We have chosen a parallel prospective cohort, natural experiment design. To our knowledge, this approach is unique in the field of evaluating a city-wide air-pollution intervention. The strengths of this approach, using a geographically distinct comparator population, include the ability to account for secular trends in air quality arising from national-level policies and to account, at least partially, for unexpected population-wide disruption, including the COVID-19 pandemic.

The use of natural experiment designs in evaluating the efficacy of population health interventions (where a randomized design is impossible) has been argued to be the only way to generate evidence to address critical questions regarding the required investment [31].

Our study collects outcomes from multiple sources (direct physiological assessment, behavior, parental and child self-report, and health-record data), permitting both triangulation across sources and elucidation of possible mechanistic pathways that influence health.

The study benefits from a high-resolution and well-validated model to predict air-pollution exposures over the study period. In London, this is supported by detailed monitoring of the criterion pollutants, as well as components (metals, polycyclic aromatic hydrocarbons) at certain research-level roadside and background sites, as summarized by the London Air Quality Network [32]. This extensive measurement infrastructure provides data to interrogate the air-quality changes that have occurred in London following the phased introduction of the ULEZ. The monitoring infrastructure in Luton is much less developed, but has evolved throughout the lifetime of the project.

The cohort provides a robust platform on which to add sub-studies, including qualitative analyses to explore behavioral influences, choices, and mechanisms, gathering additional outcomes to address additional hypotheses and potentially capturing the impact of further extensions of the ULEZ.

A potential weakness of the study is attrition of the cohort over time. To allow for attrition, a 20% annual loss of participants was factored into the power estimates used to set baseline recruitment targets [16]. We maintained an annual attrition rate of <20%, despite the total loss to follow-up being >50% by the end of data collection.

A further potential limitation of our study is the imperfect match between the intervention and comparator sites. While the populations of central London and Luton have similar demographics, air pollution and monitoring are less well matched. Luton has a similar mix of local air-pollutant sources but generally lower pollutant concentrations, especially at roadside locations. Additionally, monitoring was less comprehensive in Luton at the beginning of the study compared with London. Despite these differences, Luton served as a valuable control site for our study, providing a useful comparison to central London and allowing us to isolate the effects of traffic pollution on health outcomes.

## Can I get hold of the data? Where can I find out more?

The CHILL study is keen to collaborate with other interested researchers. We will consider all reasonable requests for data access. Requests should be sent, along with a description of the proposed project, to the corresponding author and to CHILL@qmul.ac.uk at Queen Mary University of London, London, UK.

## Ethics approval

Ethics approval for research on human participants has been given by Queen Mary University of London Research Ethics Committee (2018/08) and NHS West of Scotland Research Ethics Committee 4 (22/WS/0065). All methods were carried out in accordance with relevant guidelines and regulations. Informed-consent forms were completed by caregivers and children at home. Written informed consent was obtained from the participants’ parent/carer. Participants were also asked for their verbal assent. The study has been registered on ClinicalTrials.gov (NCT04695093, 05/01/2021).

## Supplementary Material

dyaf055_Supplementary_Data

## Data Availability

The datasets used and/or analysed during the current study are available from the corresponding author upon reasonable request.
