# Supplementary material for: Cohort Profile: The Children’s Health in London and Luton (CHILL) cohort
Source: Int J Epidemiol. 2025 May 29;54(3):dyaf055. doi: 10.1093/ije/dyaf055 (PMC12122079; doi:10.1093/ije/dyaf055)
Supplement: dyaf055_Supplementary_Data [file dyaf055_supplementary_data.docx]

**Cohort Profile: the Children’s Health in London and Luton (CHILL) cohort**

**Supplementary Material**

**Annual parental questionnaire**

Parents were asked to complete an annual questionnaire coincident with the health assessment. Data collected via these questionnaires included (*questions added after the baseline year):

- Ethnicity
- Home address and address history
- Mode of travel to school
- Exposure to second-hand cigarette smoke
- Respiratory and allergic symptoms (International Study of Asthma and Allergy in children questionnaire, ISAAC) (1)
- Health-related quality of life (Child Health Utility 9D questionnaire, CHU9D) (1)
- School absence due to ill health and consequent parental work absence
- Non-NHS health related costs
- GP details and NHS number
- Birth weight*
- Car ownership*
- Household composition*
- Use of vitamin supplements*
- COVID infection and symptoms*
- School attendance, time spent outdoors and cooking at home*

**Modelled estimates of air quality exposure**

Individual participant air pollutant exposures at the level of home address will be estimated based on high resolution monthly and annual models. Estimates will be derived from the CMAQ (Community Multiscale Air Quality Modelling System) urban model, which combines the ADMS (Advanced Dispersion Modelling System) roads model, WRF (Weather Research and Forecasting) meteorological model, and CMAQ regional scale models (2), to estimate exposures to various air pollutants, including NOx, NO_2_, O_3_, SO_2_, PM_2.5_, PM_10_, and PM components – primary and secondary inorganic and organic fractions. Exposure estimates will be produced at resolutions of up to 20m² in urban areas of the UK.

**Cognitive function and mental health assessments**

Cognitive function assessments included measurements of four cognitive domains:

- Working memory – combined scores from forward digit recall (FDR), backward digit recall (BDR), and a visuospatial memory task;
- Sensorimotor function – average scores from three tasks measuring tracking, tracing and aiming skills;
- Processing speed – single task;
- Inhibition control – single task.

The RCADs questionnaire provides measurements of overall anxiety and depression alongside sub-scale measurements for generalised anxiety, separation anxiety, social phobia, panic, obsessive/compulsive behaviour and major depression. While the SDQ questionnaire provides a measure of overall behavioural difficulties alongside sub-scale measurements for emotional, conduct and peer problems, hyperactivity, and prosocial behaviour.

**Health records data to be collected**

From primary care records and hospital episode statistics, data will be collected from birth for: general practice (GP) visits; respiratory conditions, allergies, infections including COVID-19, mental health conditions and developmental disorders; prescriptions; and hospital admissions.

**References**

1. Tsocheva I, Scales J, Dove R, et al. Investigating the impact of London’s ultra low emission zone on children’s health: children’s health in London and Luton (CHILL) protocol for a prospective parallel cohort study. BMC Pediatr. 2023 Nov 4;23(1):556
2. Beevers SD, Kitwiroon N, Williams ML, et al. Air pollution dispersion models for human exposure predictions in London. J Expo Sci Environ Epidemiol. 2013;23(6):647-53.

**Figures and Tables**

**Figure S1:** Ultra low emission zone (ULEZ) configuration during phase 1 (2019) and the subsequent extension in 2021. Panels illustrate the size of the area covered by the ULEZ from 8^th^ April 2019 and 25^th^ October 2021, with the minimum Euro emission standards for different vehicle classes illustrated, and the daily charges for non-compliant vehicles.

Figure taken from <https://www.london.gov.uk/sites/default/files/draft_london_plan_iia.pdf> (last accessed 6th September 2024)

**Figure S2:** Cohort recruitment and completeness of baseline data collection. FEV_1_, forced expiratory volume in 1 second.


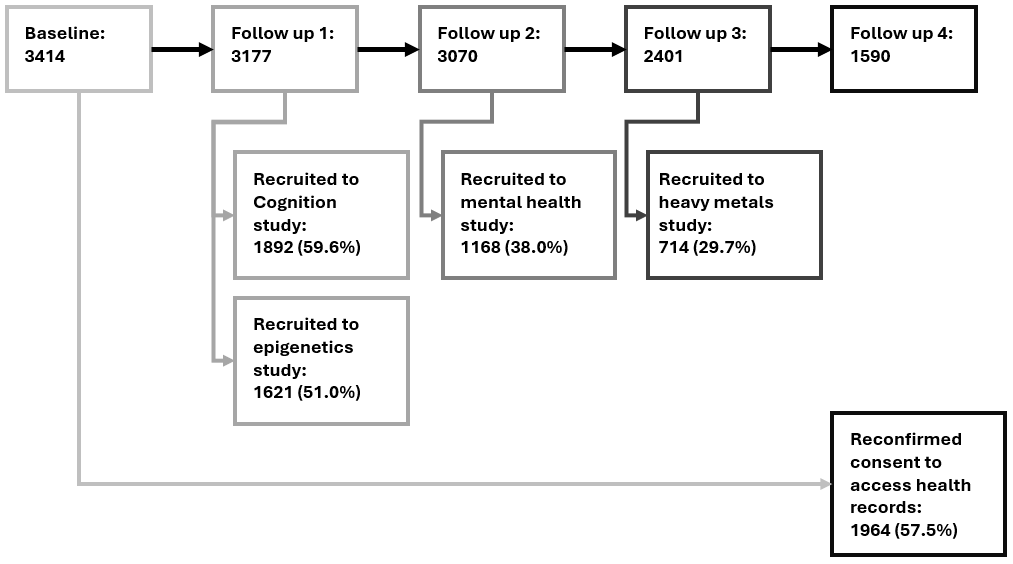


**Figure S3:** Recruitment to Children’s Health in London and Luton (CHILL) sub-studies over the first four years of follow up.

**Table S1:** Baseline characteristics of participants who were retained throughout the five-year study and those who were lost to follow up after baseline. Data are presented as mean and standard deviation (SD) for continuous variables and as frequency and % for categorical variables.

| **Characteristic** | **Retained Throughout Study** | | **Lost to Follow up** | |
| --- | --- | --- | --- | --- |
|  | London  (N = 754) | Luton  (N = 836) | London  (N = 910) | Luton  (N = 914) |
| Age – yr | 7.7±0.9 | 7.7±0.8 | 8.2±0.8 | 7.9±0.8 |
| Female sex – no. (%) | 416 (55.6) | 407 (48.7) | 503 (55.3) | 453 (49.6) |
| Height – cm | 127.4±8.0 | 126.7±7.4 | 130.8±7.8 | 128.4±7.3 |
| Weight – kg | 27.7±6.4 | 27.3±6.4 | 30.1±7.8 | 28.6±7.0 |
| BMI – kg/m^2^ | 16.9±2.5 | 16.8±2.8 | 17.4±3.1 | 17.1±2.9 |
| Ethnicity |  |  |  |  |
| White – no. (%) | 239 (31.7) | 334 (40.0) | 257 (28.2) | 329 (36.0) |
| Asian/Asian British – no. (%) | 207 (27.5) | 353 (42.2) | 162 (17.8) | 326 (35.7) |
| Black/Black British – no. (%) | 138 (18.3) | 48 (5.7) | 179 (19.7) | 84 (9.2) |
| Mixed – no. (%) | 86 (11.4) | 63 (7.5) | 138 (15.2) | 79 (8.6) |
| Other – no. (%) | 59 (7.8) | 21 (2.5) | 81 (8.9) | 25 (2.7) |
| Missing – no. (%) | 25 (3.3) | 17 (2.0) | 93 (10.2) | 71 (7.8) |
| IMD score | 3.5±1.6 | 4.1±2.1 | 3.6±1.8 | 4.0±2.0 |

**Table S2a:** Sociodemographic characteristics of recruited cohorts compared with the general populations in London and Luton. Data are percentages.

| **Demographic characteristic** | **London cohort (n=1664)** | **London population**  **(2021 Census data)** | **Luton cohort (n=1750)** | **Luton population (2021 Census data)** |
| --- | --- | --- | --- | --- |
| Female (%) | 55.2 | 51.5 (all ages) | 49.1 | 48.5 (5-9 age group) |
| Ethnicity (%) |  |  |  |  |
| White | 29.8 | 53.8 (all ages) | 37.9 | 45.2 (all ages) |
| Asian/Asian British | 22.2 | 20.7 | 38.8 | 37.0 |
| Black | 19.1 | 13.5 | 7.5 | 14.2 |
| Mixed | 13.5 | 5.7 | 8.1 | 4.3 |
| Other | 8.4 | 6.3 | 2.6 | 3.5 |
| IMD score | 3.59 | NA | 4.03 | 70 out of 317 local authorities |

IMD, index of multiple deprivation. NA, not available. Source of London demographic data:
<https://www.nomisweb.co.uk/sources/census_2021/report?compare=E12000007>

Source of London IMD data: <https://data.london.gov.uk/dataset/indices-of-deprivation>

Source of Luton data: <https://www.luton.gov.uk/Community_and_living/Lists/LutonDocuments/PDF/observatory/jsna-this-is-Luton.pdf>

**Table S2b:** Sociodemographic characteristics of the general populations in London boroughs from which the London cohort was recruited. Data are percentages.

| **Demographic characteristic** | **Islington** | **Hackney** | **Lambeth** | **Southwark** | **City of London** | **Westminster** | **Tower Hamlets** | **Camden** |
| --- | --- | --- | --- | --- | --- | --- | --- | --- |
| Female (%) | 52.3 | 51.0 | 51.5 | 51.6 | 45.0 | 51.6 | 49.8 | 52.7 |
| Ethnicity (%) |  |  |  |  |  |  |  |  |
| White | 62.2 | 53.1 | 55.0 | 51.4 | 69.4 | 55.2 | 39.4 | 59.5 |
| Asian/Asian British | 9.9 | 10.4 | 7.3 | 9.9 | 16.8 | 16.8 | 44.4 | 18.1 |
| Black | 13.3 | 21.1 | 24.0 | 25.1 | 2.7 | 8.1 | 7.3 | 9.0 |
| Mixed | 7.5 | 6.7 | 8.1 | 7.2 | 5.5 | 6.5 | 5.0 | 6.6 |
| Other | 7.1 | 8.7 | 5.7 | 6.3 | 5.6 | 13.5 | 3.9 | 6.8 |
| IMD ranking (out of 317 LAs) | 28 | 7 | 42 | 43 | 208 | 134 | 27 | 132 |
| IMD average score | 27.5 | 22 | 25.4 | 25.8 | 14.7 | 20.3 | 27.9 | 20.1 |

IMD, index of multiple deprivation. Source of London demographic data: <https://www.nomisweb.co.uk/sources/census_2021/report?compare=E12000007>

Source of London IMD data: <https://data.london.gov.uk/dataset/indices-of-deprivation>


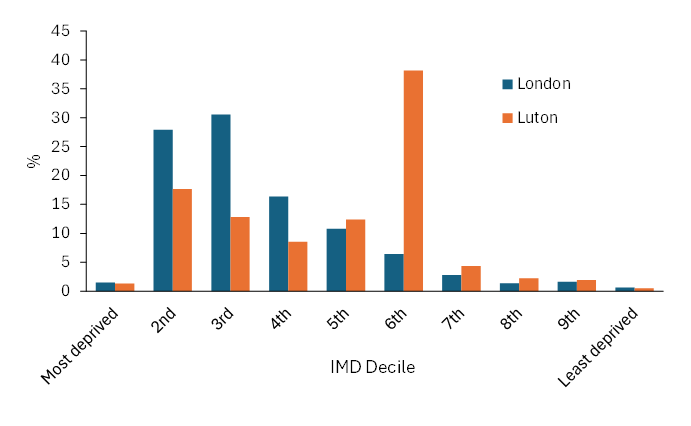


**Figure S4:** Distribution of cohort, by site, across deciles of deprivation (IMD 2019), data are percentage of participants with IMD assigned

| **Table S3**: Network monitoring sites used to calculate annual mean concentrations of criterion pollutants for baseline years of study (2018/19). | | | | | |
| --- | --- | --- | --- | --- | --- |
| **Monitoring site** | **Study site** | **Type** | **NO_2_** | **PM_2.5_** | **PM_10_** |
| Southwark, Tower Bridge Road | London | Roadside | X |  | X |
| Hackney, Old Street | London | Roadside | X | X | X |
| Westminster, Duke Street | London | Roadside | X |  |  |
| Westminster, Marylebone Road | London | Kerbside | X | X | X |
| Westminster, Cavendish Square | London | Roadside | X |  | X |
| Westminster, Oxford Street | London | Kerbside | X |  | X |
| Westminster, Horseferry Road | London | Urban background | X | X | X |
| Westminster, Covent Garden | London | Urban background | X |  |  |
| Southwark, Elephant & Castle | London | Urban background | X |  | X |
| Camden, Bloomsbury Square | London | Urban background | X | X | X |
| Luton A505 | Luton | Roadside | X |  |  |
| Luton Dunstable Road East | Luton | Roadside | X | X | X |
| Luton Airport | Luton | Urban background |  |  | X |

NO_2_, nitrogen dioxide; PM_10_, particulate matter with an aerodynamic diameter less than 10 microns; PM_2.5_, particulate matter with an aerodynamic diameter less than 10 microns. Roadside, monitoring site within 1-5m of a busy road; kerbside, monitoring site within 1m of the kerb of a busy road; urban background, monitoring site in an urban area, not close to a busy road.


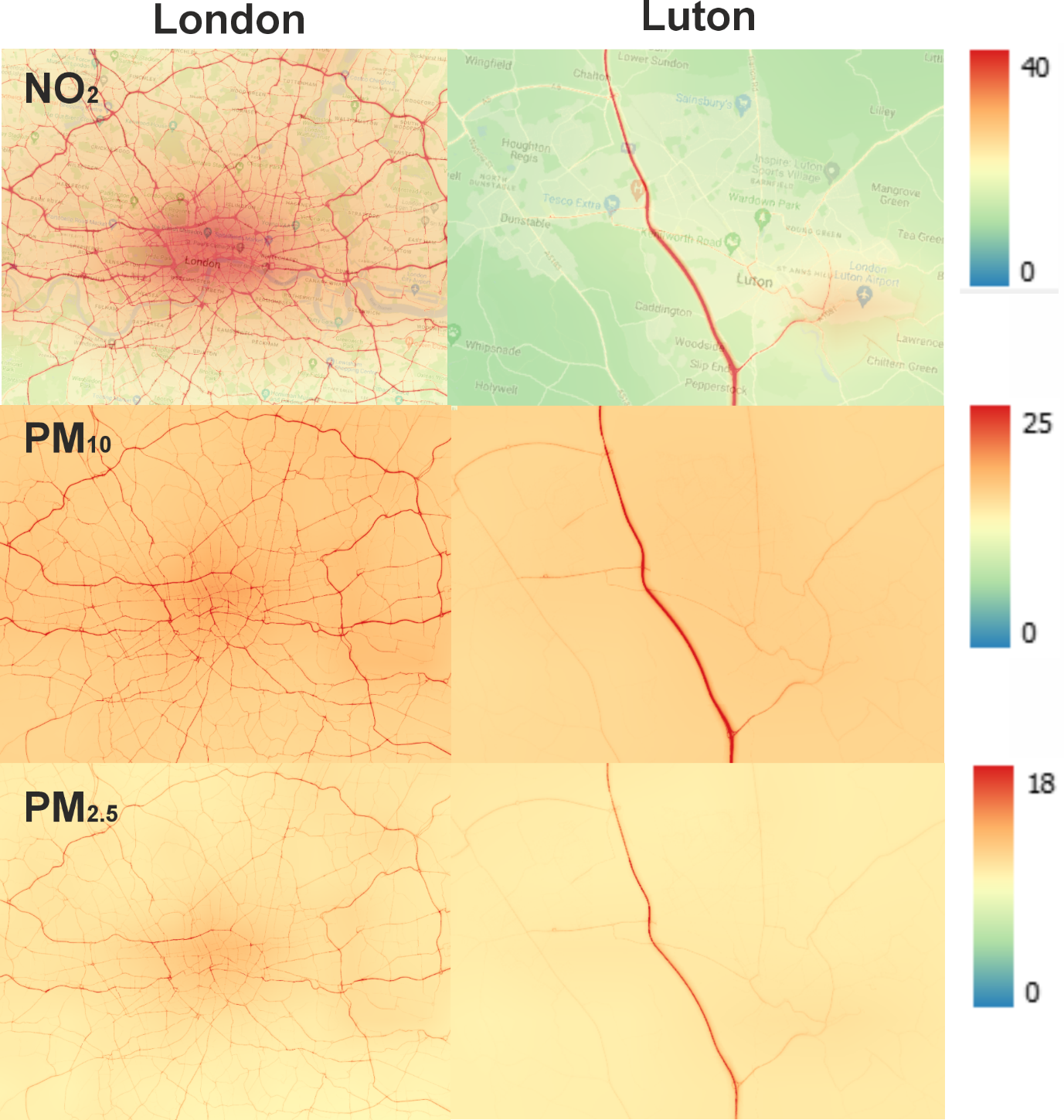


**Figure S5:** Modelled annual pollutant concentrations for nitrogen dioxide (NO_2_) and particulate matter with a diameter of less than 10 micrograms (PM_10_) and less than 2.5 micrograms (PM_2.5_) across London and Luton study sites for 2018. All concentrations expressed as µg/m^3^

| **Table S4:** Health related quality of life of primary school-aged children in London and Luton based on Child Health Utility 9D Questionnaire, data are given as n (% of returned parental questionnaires). | | | |
| --- | --- | --- | --- |
| **Domain** | **All**  **(n = 3143)** | **London**  **(n = 1486)** | **Luton**  **(n = 1657)** |
| **My child feels worried (today)** |  |  |  |
| Doesn’t feel worried | 2629 (83.6) | 1234 (83.0) | 1395 (84.2) |
| Feels a little bit worried | 262 (8.3) | 135 (9.1) | 127 (7.7) |
| Feels a bit worried | 85 (2.7) | 39 (2.6) | 46 (2.8) |
| Feels quite worried | 39 (1.2) | 16 (1.1) | 23 (1.4) |
| Feels very worried | 7 (0.2) | 2 (0.1) | 5 (0.3) |
| Missing ^a^ | 121 (3.8) | 60 (4.0) | 61 (3.7) |
| **My child feels sad (today)** |  |  |  |
| Doesn’t feel sad | 2674 (85.1) | 1266 (85.2) | 1408 (85.0) |
| Feels a little bit sad | 248 (7.9) | 110 (7.4) | 138 (8.3) |
| Feels a bit sad | 67 (2.1) | 34 (2.3) | 33 (2.0) |
| Feels quite sad | 21 (0.7) | 12 (0.8) | 9 (0.5) |
| Feels very sad | 13 (0.4) | 7 (0.5) | 6 (0.4) |
| Missing ^a^ | 120 (3.8) | 57 (3.8) | 63 (3.8) |
| **My child has pain (today)** |  |  |  |
| Doesn’t have any pain | 2651 (84.3) | 1233 (83.0) | 1418 (85.6) |
| A little bit of pain | 276 (8.8) | 147 (9.9) | 129 (7.8) |
| A bit of pain | 72 (2.3) | 37 (2.5) | 35 (2.1) |
| Quite a lot of pain | 19 (0.6) | 9 (0.6) | 10 (0.6) |
| A lot of pain | 4 (0.1) | 2 (0.1) | 2 (0.1) |
| Missing ^a^ | 121 (3.8) | 58 (3.9) | 63 (3.8) |
| **My child feels tired (today)** |  |  |  |
| Doesn’t feel tired | 1747 (55.6) | 826 (55.6) | 921 (55.6) |
| Feels a little bit tired | 920 (29.3) | 430 (28.9) | 490 (29.6) |
| Feels a bit tired | 238 (7.6) | 113 (7.6) | 125 (7.5) |
| Feels quite tired | 95 (3.0) | 46 (3.1) | 49 (3.0) |
| Feels very tired | 43 (1.4) | 22 (1.5) | 21 (1.3) |
| Missing ^a^ | 100 (3.2) | 49 (3.3) | 51 (3.1) |
| **My child feels annoyed (today)** |  |  |  |
| Doesn’t feel annoyed | 2412 (76.7) | 1115 (75.0) | 1297 (78.3) |
| Feels a little bit annoyed | 439 (14.0) | 229 (15.4) | 210 (12.7) |
| Feels a bit annoyed | 111 (3.5) | 53 (3.6) | 58 (3.5) |
| Feels quite annoyed | 41 (1.3) | 20 (1.3) | 21 (1.3) |
| Feels very annoyed | 24 (0.8) | 11 (0.7) | 13 (0.8) |
| Missing ^a^ | 116 (3.7) | 58 (3.9) | 58 (3.5) |
| **My child has problems with Schoolwork/Homework (today)** |  |  |  |
| No problems with schoolwork/homework | 2367 (75.3) | 1104 (74.3) | 1263 (76.2) |
| A few problems with schoolwork/homework | 472 (15.0) | 228 (15.3) | 244 (14.7) |
| Some problems with schoolwork/homework | 138 (4.4) | 68 (4.6) | 70 (4.2) |
| Many problems with schoolwork/homework | 45 (1.4) | 20 (1.3) | 25 (1.5) |
| Can’t do schoolwork/homework | 20 (0.6) | 12 (0.8) | 8 (0.5) |
| Missing ^a^ | 100 (3.2) | 53 (3.6) | 47 (2.8) |
| **My child has problems with Sleep (last night)** |  |  |  |
| Had no problems sleeping | 2524 (80.3) | 1171 (78.8) | 1353 (81.7) |
| Had a few problems sleeping | 383 (12.2) | 188 (12.7) | 195 (11.8) |
| Had some problems sleeping | 106 (3.4) | 56 (3.8) | 50 (3.0) |
| Had many problems sleeping | 40 (1.3) | 22 (1.5) | 18 (1.1) |
| Couldn’t sleep at all | 4 (0.1) | 3 (0.2) | 1 (0.1) |
| Missing ^a^ | 85 (2.7) | 45 (3.0) | 40 (2.4) |
| **My child has problems with Daily routine (today)** |  |  |  |
| No problems with daily routine | 2625 (83.5) | 1229 (82.7) | 1396 (84.2) |
| A few problems with daily routine | 330 (10.5) | 164 (11.0) | 166 (10.0) |
| Some problems with daily routine | 76 (2.4) | 36 (2.4) | 40 (2.4) |
| Many problems with daily routine | 23 (0.7) | 10 (0.7) | 13 (0.8) |
| Can’t do daily routine | 5 (0.2) | 2 (0.1) | 3 (0.2) |
| Missing ^a^ | 84 (2.7) | 45 (3.0) | 39 (2.4) |
| **My child can join in Activities (today)** |  |  |  |
| Can join in with any activities | 2648 (84.3) | 1240 (83.4) | 1408 (85.0) |
| Can join in with most activities | 268 (8.5) | 122 (8.2) | 146 (8.8) |
| Can join in with some activities | 95 (3.0) | 54 (3.6) | 41 (2.5) |
| Can join in with a few activities | 33 (1.0) | 21 (1.4) | 12 (0.7) |
| Can join in with no activities | 14 (0.4) | 5 (0.3) | 9 (0.5) |
| Missing ^a^ | 85 (2.7) | 44 (3.0) | 41 (2.5) |
| **Health Related Quality of Life (Utility Score)** ^b^ |  |  |  |
| N (%) | 2968 (94.4) | 1400 (94.2) | 1568 (94.6) |
| Score based on Child Health Utility 9D Questionnaire, mean ± SD | 0.96 ± 0.06 | 0.96 ± 0.05 | 0.96 ± 0.06 |
| SD, standard deviation; ^a^ not reported or not known; ^b^ calculated for respondents with all domains reported. | | | |

| **Table S5:** Costs incurred due to child’s respiratory illness over 12 months prior to baseline health assessment. Data are given as n (% of returned parental questionnaires) for categorical variables and as mean ± standard deviation for continuous variables. | | | |
| --- | --- | --- | --- |
| **Cost incurred** | **All**  **(n = 3143)** | **London**  **(n = 1486)** | **Luton**  **(n = 1657)** |
| **Medication/equipment costs** |  |  |  |
| Yes | 1411 (44.9) | 615 (41.4) | 796 (48.0) |
| No | 1364 (43.4) | 660 (44.4) | 704 (42.5) |
| Missing^a^ | 368 (11.7) | 211 (14.2) | 157 (9.5) |
| Mean ± SD reported costs, per child | 69.9 ± 277.2 | 65.8 ± 137.1 | 73.4 ± 353.8 |
| **Childcare costs** |  |  |  |
| Yes | 108 (3.4) | 56 (3.8) | 52 (3.1) |
| No | 2711 (86.3) | 1266 (85.2) | 1445 (87.2) |
| Missing^a^ | 324 (10.3) | 164 (11.0) | 160 (9.7) |
| Mean ± SD reported costs, per child | 180.5 ± 172.2 | 188.3 ± 169.6 | 164.3 ± 182.3 |
| SD = standard deviation; ^a^ not reported or not known | | | |

| **Table S6:** Parental work absence due to child’s illness over 12 months prior to baseline health assessment. Data are given as n (% of returned parental questionnaires) for categorical variables and as mean ± standard deviation for continuous variables. | | | |
| --- | --- | --- | --- |
| **Work absence** | **All**  **(n = 3143)** | **London**  **(n = 1486)** | **Luton**  **(n = 1657)** |
| **Work absence for any child illness** |  |  |  |
| Yes | 709 (22.6) | 355 (23.9) | 354 (21.4) |
| No | 1737 (55.3) | 784 (52.8) | 953 (57.5) |
| Missing^a^ | 697 (22.2) | 347 (23.4) | 350 (21.1) |
| Mean ± SD days of absence, per child^b^ | 4.19 ± 5.32 | 4.26 ± 5.87 | 4.11 ± 4.72 |
| **Work absence for child respiratory illness** |  |  |  |
| Yes | 340 (10.8) | 193 (13.0) | 147 (8.9) |
| No | 2026 (64.5) | 903 (60.8) | 1123 (67.8) |
| Missing^a^ | 777 (24.7) | 390 (26.2) | 387 (23.4) |
| Mean ± SD days of absence, per child^b^ | 3.90 ± 4.73 | 3.61 ± 3.42 | 4.28 ± 6.01 |
| SD, standard deviation; ^a^ not reported or not known; ^b^ across both/any parents who reported the number of days of work absence | | | |
